# High sensitivity bolometers based on metal nanoantenna dimers with a nanogap filled with vanadium dioxide

**DOI:** 10.1038/s41598-021-95429-1

**Published:** 2021-08-05

**Authors:** Dukhyung Lee, Dasom Kim, Dai-Sik Kim, Hyeong-Ryeol Park, Changhee Sohn, Seon Namgung, Kunook Chung, Young Chul Jun, Dong Kyun Kim, Hyuck Choo, Young-Geun Roh

**Affiliations:** 1 Department of Physics and Quantum Photonics Institute, Ulsan National Institute of Science and Technology (UNIST), Ulsan, 44949 Republic of Korea; 2 School of Materials Science and Engineering, Ulsan National Institute of Science and Technology (UNIST), Ulsan, 44919 Republic of Korea; 3 Samsung Advanced Institute of Technology, Samsung Electronics, Suwon, 16678 Republic of Korea

**Keywords:** Optical sensors, Sub-wavelength optics, Nanophotonics and plasmonics

## Abstract

One critical factor for bolometer sensitivity is efficient electromagnetic heating of thermistor materials, which plasmonic nanogap structures can provide through the electric field enhancement. In this report, using finite element method simulation, electromagnetic heating of nanorod dimer antennas with a nanogap filled with vanadium dioxide (VO_2_) was studied for long-wavelength infrared detection. Because VO_2_ is a thermistor material, the electrical resistance between the two dimer ends depends on the dimer’s temperature. The simulation results show that, due to the high heating ability of the nanogap, the temperature rise is several times higher than expected from the areal coverage. This excellent performance is observed over various nanorod lengths and gap widths, ensuring wavelength tunability and ultrafast operating speed, thereby making the dimer structures a promising candidate for high sensitivity bolometers.

## Introduction

Bolometers are devices absorbing thermal radiation and translating the temperature increase into an electrical resistance change by which the thermal status of an object can be measured^1–3^. A key factor of bolometer sensitivity is electromagnetic heating efficiency of thermistor materials in the designed wavelength range. In the recent decade, efficient electromagnetic heating has been demonstrated in a variety of plasmonic structures and metamaterials, paving the way for advanced bolometers^4–7^. A noteworthy structure is the plasmonic nanogap that resonantly confines electromagnetic wave inside the gap resulting in a strong electric field enhancement, and thus efficient heating of gap material. Over various spectral ranges from microwave to visible light, enormous intensity enhancement factors over 10^4^ have been reported using plasmonic nanogap structures^8–13^.

Vanadium dioxide (VO_2_) is widely used as a thermistor material in bolometers due to its high negative temperature coefficient of resistance (TCR) below − 0.02 K^−1^ at the room temperature which is about 5–10 times better than the TCR of most metals^14–16^. Therefore, it is desirable to integrate VO_2_ with plasmonic nanogap structures for high performance bolometers. A recently developed fabrication technique called atomic layer lithography suggests a possibility to accomplish such integration. In this technique, a dielectric material that is grown by atomic layer deposition (ALD) comprises the gap material, and accordingly, the gap width defined by the dielectric thickness can be sub-10 nm with Ångström resolution, maximizing the field enhancement factor^8–11,17,18^. It was demonstrated that various types of antennas can be combined with nanogap structures by using atomic layer lithography^9,17^. Because ALD of VO_2_ has been already developed, enabling sub-10 nm thickness and a roughness under 1 nm^19,20^, atomic layer lithography can be applied straightforwardly to the fabrication of VO_2_ nanogap structures.

Under these recent developments, a theoretical assessment on the bolometric response of VO_2_ nanogap structures would be valuable, keeping up with the advances in fabrication techniques discussed above. In this paper, we conducted finite element method simulation to calculate electromagnetic heating of the gold nanorod dimer with a VO_2_ nanogap in the long-wavelength infrared (LWIR) range which is the most important range for bolometers designed to detect room temperature objects^3,21^. Our result shows that, when compared to a perfectly absorbing structure of the same thermal time constant, the temperature increase of the VO_2_ nanogap structure is about 18 times at the resonance, implying a high sensitivity of the end-to-end resistance to the LWIR irradiation. We also investigated the absorption and the field enhancement in the VO_2_ nanogap to reveal the mechanism of the excellent bolometer performance and demonstrated resonance tuning and ultrafast pulse detection by changing the nanorod length and the gap width.

## Results and discussion

**Figure 1:**
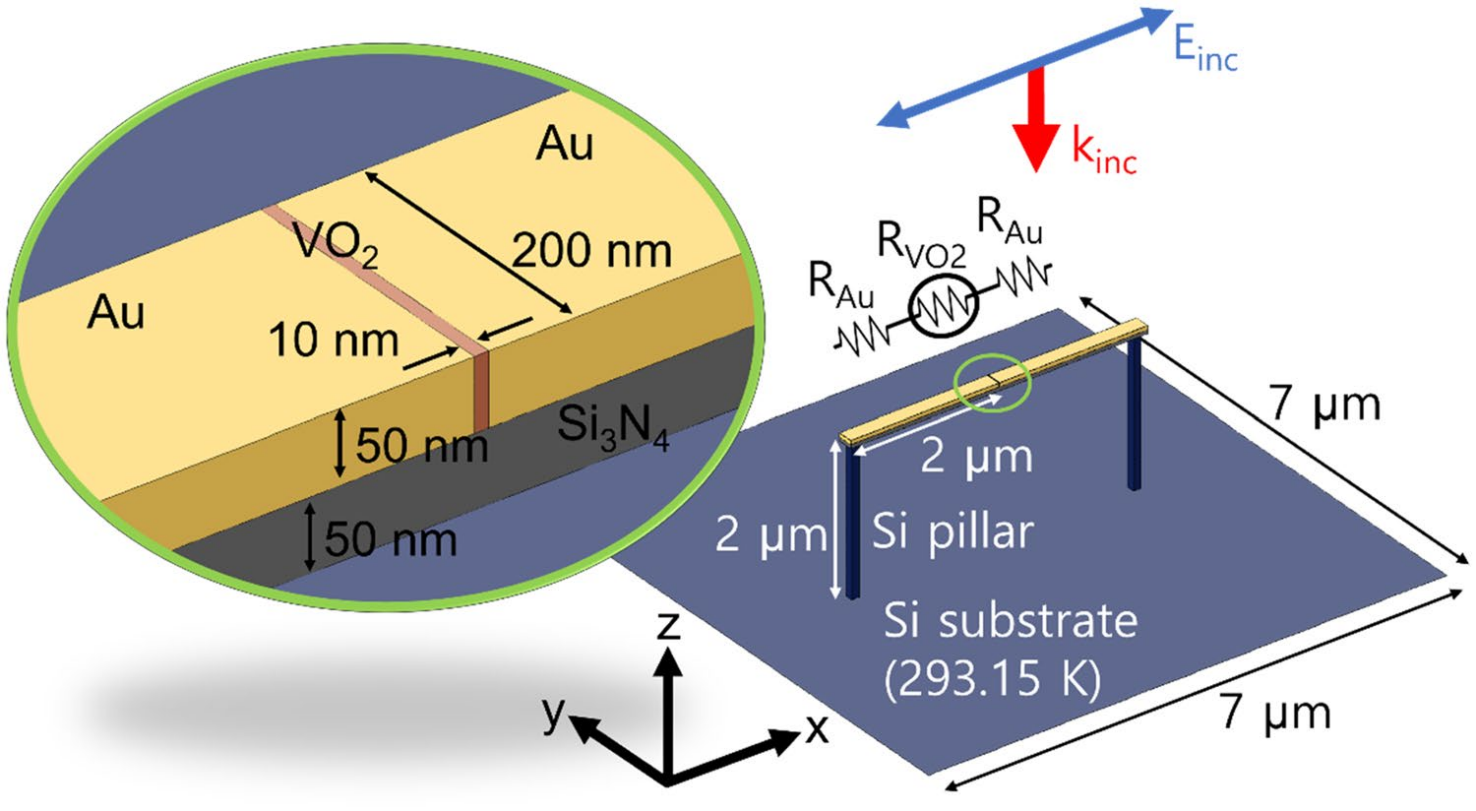
Schematics of the unit cell structure under study. VO_2_ (red) fills the gap between the two gold rods (yellow). The dimer is supported by a Si_3_N_4_ layer (gray) and connected to a Si substrate by two Si pillars (dark blue). The substrate is assumed to be a heat reservoir maintained at 293.15 K. Red and blue arrows represent the incident direction and the polarization of LWIR, respectively.

We start this section by a brief explanation on the structure and the conditions we studied. Assuming high density array applications, a unit cell with a periodicity of 7 μm × 7 μm was considered. Figure 1 illustrates the unit cell for a gold dimer structure where the gap of 10 nm-width is filled with VO_2_. The length of the dimer was set to have a resonance in the LWIR range. In order to focus on the performance assessment of the dimer structure itself, we simplified supporting structures with a Si_3_N_4_ membrane and two Si pillars. The Si pillars thermally and electrically connect the dimer structure (Fig. S1 for the detailed pillar geometry) to a Si substrate which acts as a heat reservoir maintained at the room temperature, 293.15 K. Readout circuits can be formed on/inside the Si substrate but were omitted from this simulation for the sake of brevity. We also assumed convective heat dissipation with a typical transfer coefficient of 5 W/(m^2^K) for ambient air^22^. Setting the initial temperature at 293.15 K, we conducted electromagnetic heating simulation using a commercial finite element method software (COMSOL Multiphysics 5.5) within the LWIR range from 8 to 14 μm with a step size of 0.05 μm. Incident light was assumed to be a *x*-polarized plane wave propagating in the − *z* direction with a power of 5 nW/μm^2^ (245 nW per unit cell). Periodic conditions were applied to the unit cell lateral boundaries. After obtaining the average temperatures of the gap and the rods in the simulation, the dimer resistance between the two ends was calculated as the sum of the three resistors shown in the circuit diagram in Fig. 1 exploiting the temperature dependent electrical conductivities of gold and VO_2_. Details of the material properties used in the calculation are presented in the "Methods" section.

**Figure 2:**
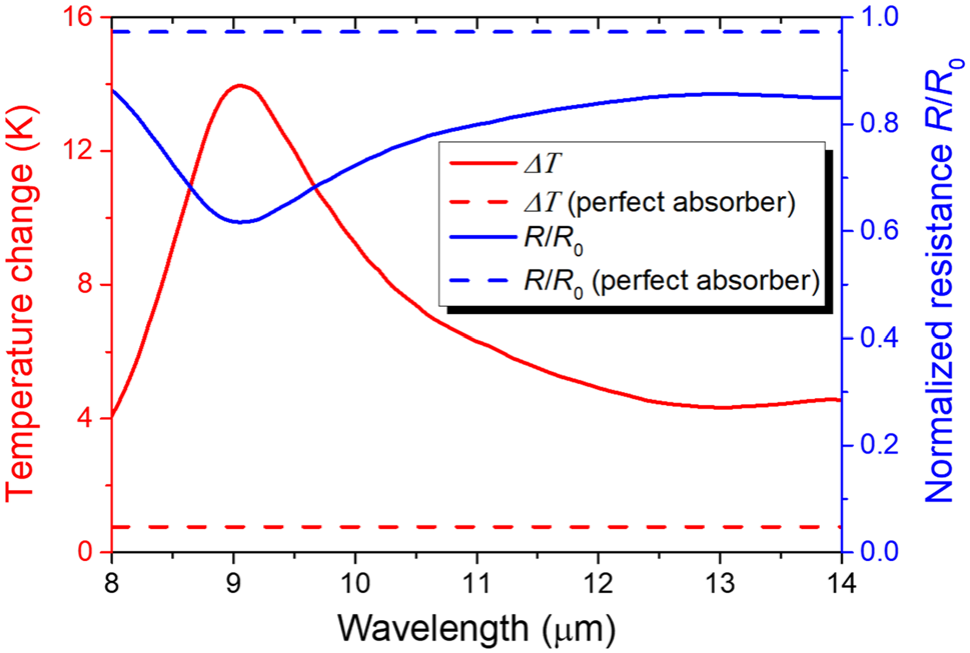
Average temperature change *ΔT* of the VO_2_ gap in the dimer under the 5 nW/μm^2^ LWIR irradiation (red solid line) and the corresponding end-to-end resistance normalized by the room temperature resistance *R*/*R*_0_ (blue solid line). For comparison, average temperature change of a perfect absorbing VO_2_ film with the same thermal time constant (red dashed line) and the film’s normalized resistance (blue dotted line) are also displayed.

To evaluate the bolometric response of the dimer, we calculated the temperature increase and the corresponding resistance when the incident LWIR power is 5 nW/μm^2^. As shown in Fig. 2, the VO_2_ gap temperature increases by more than 4 K across the whole LWIR range. Especially, at the resonance wavelength of 9.05 μm, the temperature increases by nearly 14 K. Advantage of the dimer structure becomes evident when compared with a suspended perfect absorbing film which can be regarded as a simplified ideal of conventional optical cavity based bolometers^3,21^. For the comparison, we simulated temperature increase of a 50 nm-thick VO_2_ film with the same unit cell dimension assuming all the incident energy is converted to heat in the film. The thermal time constant, or the ratio of the heat capacity of the suspended structure to the thermal conductance of the supporting pillars, was set to 38.2 μs which is equivalent to that of the dimer structure (Fig. S2 for the simulation details). The simulation result for the perfect absorber is displayed as the red dashed line in Fig. 2 indicating that the VO_2_ film temperature increases by only 0.77 K. Thus, the temperature increase of the dimer’s VO_2_ gap is at least 5 times larger than that of the perfect absorber throughout the LWIR range and reaches about 18 times at the resonance.

The dimer’s normalized resistance shown as the blue solid line in Fig. 2 verifies the measurable resistance decrease. At the resonance, the dimer’s resistance drops almost by 40%. This is because, although the width is only 10 nm, the VO_2_ nanogap determines most of the total resistance. The resistance of the VO_2_ nanogap drops as the temperature rises while the resistance of the gold parts constitutes a negligible portion. In contrast, the perfect absorber’s resistance was calculated to decrease by only about 3%.

**Figure 3:**
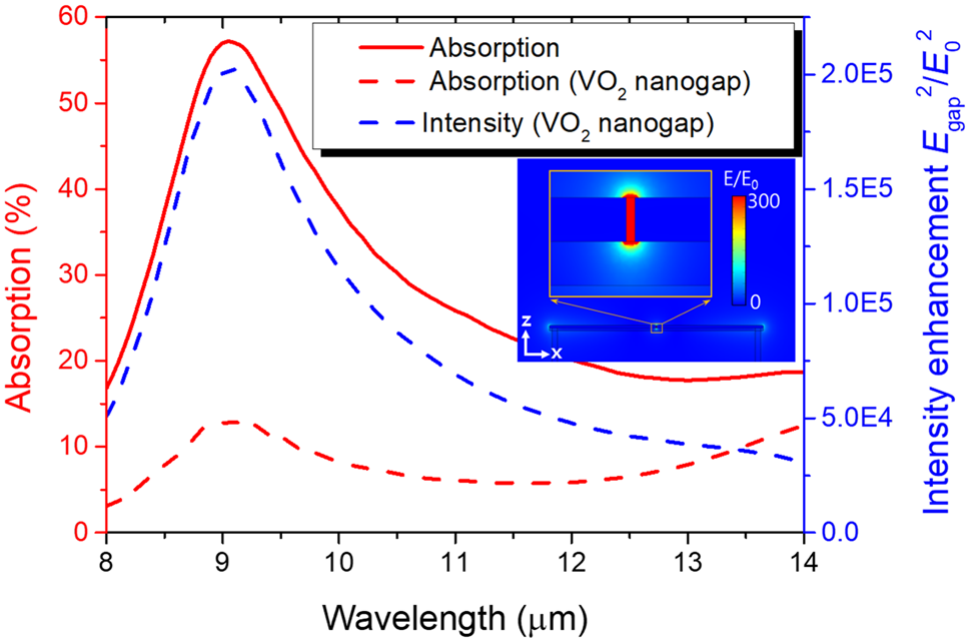
Absorption of the unit cell (red solid line) and of only the VO_2_ nanogap region in the unit cell (red dashed line) and the electric field intensity enhancement in the VO_2_ nanogap (blue dashed line). The inset illustrates the cross-sectional electric field distribution at the resonance wavelength, 9.05 μm.

We investigated electromagnetic absorption of the dimer in the LWIR range to explain the efficient electromagnetic heating described above. Spectra of the total absorption and the absorption in the nanogap are displayed as the solid and dashed red lines in Fig. 3. Although the areal coverage of the structure is only 1.6% (= (4.01 μm × 200 nm)/(7 μm)^2^), it absorbs 57% at the resonance and more than 16% over the whole LWIR range. In this absorption, the VO_2_ nanogap makes a significant contribution. At the resonance, 13% absorption occurs in the nanogap whose areal coverage is only 0.0041% (= (10 nm × 200 nm)/(7 μm)^2^). The contribution of the nanogap is more evident in the red side of the resonance, where the extinction coefficient of VO_2_ increases, leading to a rebound of the total absorption.

This enormous absorption in the VO_2_ nanogap is attributed to the electric field enhancement by the capacitive charging of the nanogap structure^23^. The intensity enhancement, that is the electric field intensity in the nanogap normalized by the incident intensity, is plotted as the blue dashed line in Fig. 3. At the resonance, the field intensity is enhanced by five orders of magnitude. The cross-sectional electric field distribution at the resonance (inset in Fig. 3) clearly displays that the enhancement is concentrated at the vicinity of the gap where charges are accumulated.

**Figure 4:**
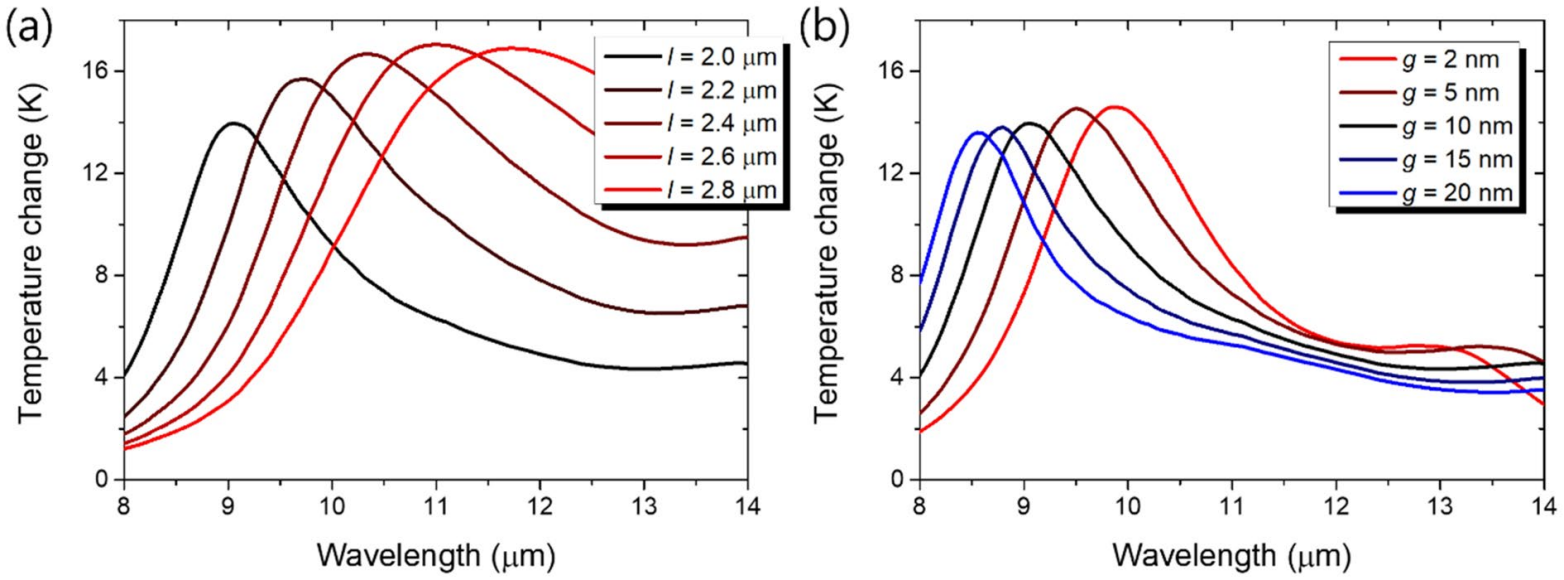
Temperature changes of the VO_2_ gap under the 5 nW/μm^2^ LWIR irradiation (**a**) for various rod lengths *l* from 2.0 to 2.8 μm and (**b**) for various gap widths *g* from 2 to 20 nm. Other parameters are the same as in Fig. 1.

We also studied wavelength tunability of the dimer structure which is important for performance optimization. Temperature changes for various nanorod lengths and gap widths are plotted in Fig. 4. As expected from the hybridization of plasmon modes^24^, longer nanorod lengths and narrower gap widths leads to redshift of the resonance. Since the heating efficiency is maintained about the same level, both the geometrical parameters can be exploited for wavelength tuning.

**Figure 5:**
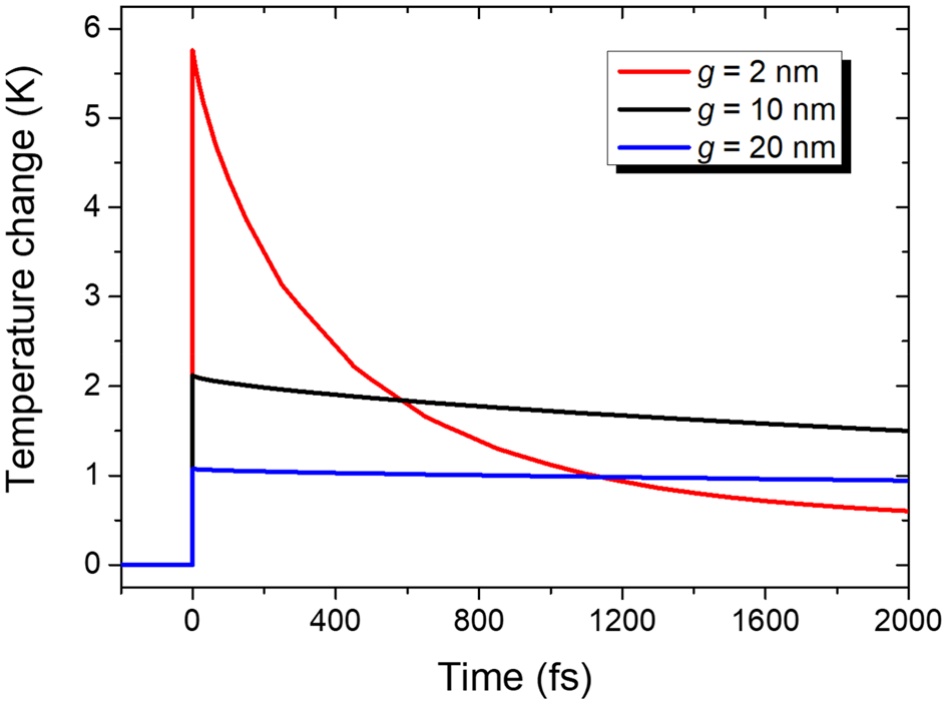
Time evolutions of the VO_2_ nanogap temperatures for gap widths *g* of 2, 10, and 20 nm. It is assumed that LWIR irradiation of respective resonant wavelengths is instantaneously incident at 0 fs with an energy of 5 fJ per unit cell.

The high heating efficiency even for the gap width of 2 nm suggests that a substantial amount of absorption still occurs in the more compact VO_2_ volume. Indeed, the 2 nm-gap absorbs 7.3% at the resonance, implying that absorption in nanogap decreases only by less than the half while gap width is reduced to one fifth compared to the case of the 10 nm-gap. This absorption concentration into the thermistor region can be especially favorable for pulse detection. To demonstrate this point clearly, we studied time evolution of a VO_2_ nanogap temperature and its width dependence assuming that resonant LWIR with a pulse energy of 5 fJ per unit cell is incident instantaneously at time zero. For simplicity and ease of simulation, we first obtained spatial absorption density distribution with a continuous wave scheme and converted it to temperature distribution at the time zero. Then, a heat transfer simulation was conducted to obtain the time evolution. Figure 5 shows that higher temperature changes and faster thermal relaxation are achieved in narrower gaps owing to the smaller thermal capacity of the gap. Although complete thermal relaxation requires several times the thermal time constant of 38.2 μs, which is determined by heat loss through the Si pillars, partial thermal relaxation by heat transfer to the gold and Si_3_N_4_ parts can be accomplished on a picosecond scale as in the case of 2 nm-width, enabling ultrafast operation for pulse detection.

## Conclusion

The nanorod dimer structure containing a VO_2_ nanogap is demonstrated to absorb LWIR radiation efficiently, exceeding the areal coverage. The VO_2_ nanogap provides, along with the high negative TCR, an enormous electromagnetic absorption by virtue of the capacitive charging, making the dimer structure attractive in bolometer applications. Because the resonance wavelength and the thermal relaxation are easily tunable by varying the nanorod length and the gap width, the dimer design is applicable in various spectral ranges and operating speeds. This study shows that VO_2_ nanogap structures fabricated by atomic layer lithography can be used to achieve high performance in various bolometer applications, providing high heating efficiency and a small areal footprint particularly suitable for high sensitivity ultrafast microbolometer arrays.

## Methods

For the electromagnetic heating simulation, both the optical and thermal properties of VO_2_ and gold should be specified. The optical properties were specified by the tabulated refractive indexes of a 70 nm-thick VO_2_ film^25^, a 437 nm-thick Si_3_N_4_ film^26^, and a Si crystal^27^, and by the tabulated dielectric function of a 200 nm-thick evaporated gold film^28^. The thermal conductivity, heat capacity, and density of gold were set to 317.8 W/(m·K), 128.4 J/(kg·K), and 19,280 kg/m^3^, respectively^29,30^. Heat capacity and density were 760 J/(kg·K) and 3190 kg/m^3^ for Si_3_N_4_^31^, and 690.4 J/(kg·K) and 2329 kg/m^3^ for Si^32^. The thermal conductivities of Si_3_N_4_ and Si were set to 0.15 and 1.0 W/(m·K), respectively, according to the measurements on thin films^33^. For the thermal properties of VO_2_, we used data measured at 291 K where the thermal conductivity, heat capacity, and density are 4.4 W/(m·K), 647.5 J/(kg·K), and 4340 kg/m^3^, respectively^34^. Note that we assumed the thermal properties to be constant because they vary by less than 10% over the simulated temperature range.

After the simulation, temperature dependent electrical resistance of the VO_2_ gap was calculated using a model for dc electric conductivity given by J. Ordonez-Miranda et al.^35^. The model is based on an effective medium theory and the conductivities of metallic and insulating domains are 84,175.1 S/m and 4.86 × 10^6^ exp(− 3136.1/*T*) S/m, respectively, in the model. Resistance of the gold nanorods was calculated assuming the resistivity and the temperature coefficient of 2.44 × 10^–8^ Ω·m and 3.4 × 10^–3^ K^-1^, respectively^36^.

## Supplementary Information

Supplementary Information.
