# Supplementary material for: High sensitivity bolometers based on metal nanoantenna dimers with a nanogap filled with vanadium dioxide
Source: Sci Rep. 2021 Aug 5;11:15863. doi: 10.1038/s41598-021-95429-1 (PMC8342706; doi:10.1038/s41598-021-95429-1)
Supplement: Supplementary file 1 — Supplementary Information. [file 41598_2021_95429_MOESM1_ESM.docx]

**Supporting information for**

**High sensitivity bolometers based on metal nanoantenna dimers with a nanogap filled with vanadium dioxide**

Dukhyung Lee^1^, Dasom Kim^1^, Dai-Sik Kim^1^, Hyeong-Ryeol Park^1^, Changhee Sohn^1^, Seon Namgung^1^, Kunook Chung^1^, Young Chul Jun^2^, Dong Kyun Kim^3^, Hyuck Choo^3^ & Young-Geun Roh^3^

^1^Department of Physics and Quantum Photonics Institute, Ulsan National Institute of Science and Technology (UNIST), Ulsan 44949, Republic of Korea.

^2^School of Materials Science and Engineering, Ulsan National Institute of Science and Technology (UNIST), Ulsan 44919, Republic of Korea.

^3^Samsung Advanced Institute of Technology, Samsung Electronics, Suwon 16678, Republic of Korea.

[hyung0624@unist.ac.kr](mailto:hyung0624@unist.ac.kr)

**Figure S1. Geometrical details of the Si pillars.**


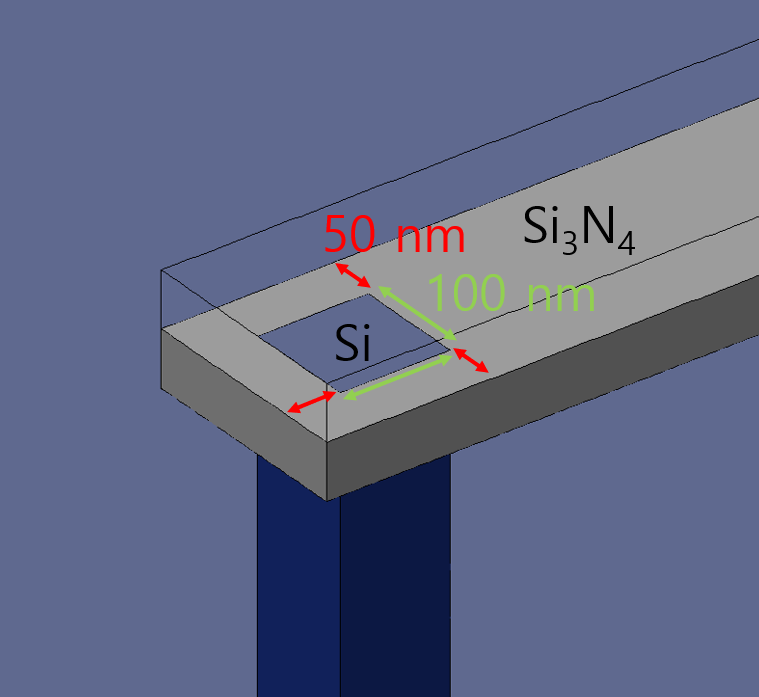


The Si pillars have the cross-sectional area of 100 nm × 100 nm. The pillars are in contact with the gold nanorods, passing through the Si_3_N_4_ layer with the margin of 50 nm. In this figure, the gold part was set to be transparent showing only the edges for clarity.

**Figure S2. Simulation setting of the perfect absorber.**


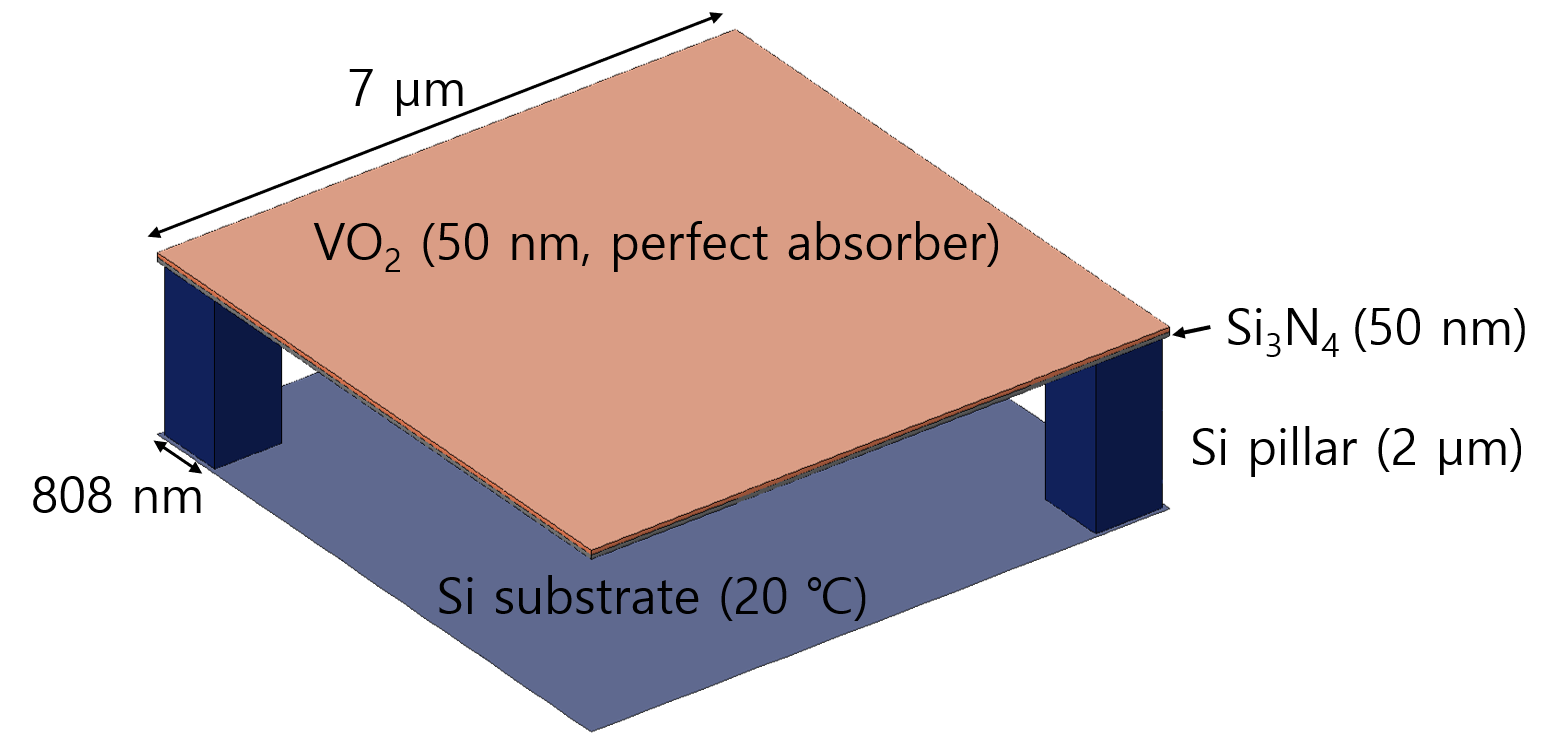


It was assumed that 5 nW/μm^2^ incident infrared light is perfectly absorbed by the VO_2_ layer of the unit cell dimension. Accordingly, we conducted a heat transfer simulation regarding the VO_2_ layer as a heat source of 10^11^ W/m^3^ (= (5 nW/μm^2^)/50 nm). Cross-sectional area of a Si pillar was determined to be 808 nm × 808 nm which makes the time constant *τ* = *C*/*G* (*C*: Heat capacity of the suspended structure, *G*: Thermal conductance of the pillars) of the perfect absorbing structure almost equal to the time constant of the dimer structure. All material properties were the same as in the nanoantenna dimer simulation.
